# Low tidal volume ventilation for patients undergoing laparoscopic surgery: a secondary analysis of a randomised clinical trial

**DOI:** 10.1186/s12871-023-01998-1

**Published:** 2023-03-07

**Authors:** Dharshi Karalapillai, Laurence Weinberg, Ary Serpa Neto, Philip J. Peyton, Louise Ellard, Raymond Hu, Brett Pearce, Chong O. Tan, David Story, Mark O’Donnell, Patrick Hamilton, Chad Oughton, Jonathan Galtieri, Anthony Wilson, Grace Liskaser, Ajay Balasubramaniam, Glenn Eastwood, Rinaldo Bellomo, Daryl A. Jones

**Affiliations:** 1 Department of Anesthesia, Austin Hospital, Studley Rd, Heidelberg, Melbourne, Victoria 3084 Australia; 2 Department of Intensive Care, Austin Hospital, Melbourne, Victoria Australia; 3 Australian and New Zealand Intensive Care Research Centre, School of Public Health and Preventive Medicine, Monash University, Melbourne, Victoria Australia; 4 Department of Critical Care Medicine, The University of Melbourne, Melbourne, Victoria Australia

**Keywords:** Low tidal volume ventilation, Laparoscopic surgery, Post-operative pulmonary complications

## Abstract

**Background:**

We recently reported the results for a large randomized controlled trial of low tidal volume ventilation (LTVV) versus conventional tidal volume (CTVV) during major surgery when positive end expiratory pressure (PEEP) was equal between groups. We found no difference in postoperative pulmonary complications (PPCs) in patients who received LTVV. However, in the subgroup of patients undergoing laparoscopic surgery, LTVV was associated with a numerically lower rate of PPCs after surgery. We aimed to further assess the relationship between LTVV versus CTVV during laparoscopic surgery.

**Methods:**

We conducted a post-hoc analysis of this pre-specified subgroup. All patients received volume-controlled ventilation with an applied PEEP of 5 cmH_2_O and either LTVV (6 mL/kg predicted body weight [PBW]) or CTVV (10 mL/kg PBW). The primary outcome was the incidence of a composite of PPCs within seven days.

**Results:**

Three hundred twenty-eight patients (27.2%) underwent laparoscopic surgeries, with 158 (48.2%) randomised to LTVV. Fifty two of 157 patients (33.1%) assigned to LTVV and 72 of 169 (42.6%) assigned to conventional tidal volume developed PPCs within 7 days (unadjusted absolute difference, − 9.48 [95% CI, − 19.86 to 1.05]; *p* = 0.076). After adjusting for pre-specified confounders, the LTVV group had a lower incidence of the primary outcome than patients receiving CTVV (adjusted absolute difference, − 10.36 [95% CI, − 20.52 to − 0.20]; *p* = 0.046).

**Conclusion:**

In this post-hoc analysis of a large, randomised trial of LTVV we found that during laparoscopic surgeries, LTVV was associated with a significantly reduced PPCs compared to CTVV when PEEP was applied equally between both groups.

**Trial registration:**

Australian and New Zealand Clinical Trials Registry no: 12614000790640.

**Supplementary Information:**

The online version contains supplementary material available at 10.1186/s12871-023-01998-1.

## Introduction

There is growing interest in the role of low tidal volume ventilation (LTVV) during major surgery for the prevention of postoperative pulmonary complications (PPCs) [1–11]. However, major trials of LTVV in abdominal surgery have focused on patients undergoing major open abdominal surgery or a mixture of open and laparoscopic abdominal surgeries [1, 2]. The inclusion of such heterogeneous groups may be a problem. Laparoscopic abdominal surgery may carry unique and relevant features. For example, the physiological effects of the pneumoperitoneum, differences in patient position during surgery, postoperative pain and a lower rate of complications may influence the relationship between LTVV and PPCs in this group and the ability to detect PPCs [12–20]. Regrettably, to date, studies of intraoperative LTVV, during laparoscopic surgery, have been limited in size, focused specifically on physiological outcomes or have been confined to specific operative procedures. Moreover, they have also involved multiple ventilatory interventions with variable positive end-expiratory pressure (PEEP) settings and recruitment manoeuvres [21–24].

We recently reported the results for a large randomized controlled trial of LTVV versus conventional tidal volume during major surgery when PEEP was equal between groups. We found no difference in PPCs in patients who received LTVV, but in a subgroup of patients undergoing laparoscopic surgery LTVV was associated with a numerically lower rate of PPCs within 7 days [1]. It is unclear whether these findings indicate genuine effect of LTVV or are accounted for by chance baseline imbalances or other confounding factors that may affect this outcome.

Therefore, we conducted a post-hoc analysis using data from patients undergoing laparoscopic surgery who were included in the original trial [1]. We hypothesised that, after adjusting for confounding factors, intraoperative LTVV would be associated with a significantly reduced incidence of PPCs within 7 days of surgery.

## Materials and methods

### Study design

As part of an investigator-initiated, assessor-blinded, single centre, randomised clinical trial, we conducted a post-hoc analysis of the pre-specified subgroup of patients who underwent laparoscopic surgery. The protocol, statistical analysis plan and primary trial have been published [25].

### Ethics

The local ethics committee approved the study (HREC approval number HREC/14/Austin260). Written informed consent was obtained from all participating patients. This study was performed in accordance with the Declaration of Helsinki. The primary trial was registered with the ANZCA clinical trials network (ACTRN12614000790640).

### Patients

Patients were included in the primary trial if they were older than 40, scheduled to have major surgery of expected duration > 2 hours and anticipated to receive invasive arterial pressure monitoring as part of their routine care. Patients were excluded if they were pregnant; scheduled to have cardiac, thoracic or intracranial neurological surgery; or if they had been previously enrolled in the trial. The patients undergoing laparoscopic surgery included in this analysis comprise a pre-specified subgroup defined prior to randomisation [1, 25].

### Intervention

All patients received volume-controlled ventilation with an applied PEEP of 5 cmH_2_O. Immediately following randomisation, patients were assigned to receive either LTVV (6 mL/kg predicted body weight [PBW]) or conventional tidal volume ventilation (CTVV) (10 mL/kg PBW). PBW was calculated as follows:

$$\textrm{Male}\ \textrm{PBW}=50+0.91\ast \left(\textrm{height}\ \left[\textrm{cm}\right]-152.4\right)$$

$$\textrm{Female}\ \textrm{PBW}=45.5+0.91\ast \left(\textrm{height}\ \left[\textrm{cm}\right]-152.4\right)$$

The tidal volume and PEEP were maintained for the duration of the surgical procedure. All cases were performed under the supervision or direct care of a specialist anaesthetist. Participants underwent intravenous induction, neuromuscular blockade and endotracheal intubation, and a volatile agent was used to maintain anaesthesia. Apart from tidal volume and PEEP, other aspects of clinical care, including PaCO_2_ and oxygenation targets (SpO_2_ and PaO_2_), were administered at the discretion of the treating anaesthetist. In addition, the inspired fraction of oxygen (FiO_2_), respiratory rate, anaesthesia technique, fluid management, use of vasoactive drugs, analgesia plan, prophylactic antibiotics and anti-emetic agents were also administered at the discretion of the treating anaesthetist.

### Data collection

We used a standardised case report form for data collection. Intraoperatively, we collected all ventilatory data and vital signs prospectively as the lowest and/or highest values during the procedure. The research staff collected all data directly from the clinical chart source data. Until postoperative day seven or hospital discharge (whichever came first), the trial research team assessed all patients daily. Research staff blinded to the intraoperative intervention collected information regarding the clinical outcomes. After the first 7 days (if the patient was still in hospital), we retrieved additional data from the electronic medical record.

### Outcomes

As previously reported in our primary trial, the primary outcome for this analysis was the incidence of a composite of PPCs, defined as present if any component developed within the first 7 days after surgery. These complications included pneumonia, bronchospasm, atelectasis, pulmonary congestion, respiratory failure, pleural effusion or pneumothorax, or unplanned requirement for postoperative mechanical ventilation, continuous positive airway pressure or non-invasive or invasive ventilation (see eTable 1 in Online Supplement). The diagnoses of atelectasis, pleural effusion and pneumothorax were based on chest x-rays and adjudicated by assessors blinded to study group allocation [1].

The secondary outcomes were 1) incidence of PPCs during hospital stay, 2) incidence of pulmonary embolism, 3) incidence of acute respiratory distress syndrome, 4) incidence of systemic inflammatory response syndrome, 5) incidence of sepsis, 6) incidence of acute kidney injury, 7) incidence of wound infection (superficial or deep), 8) rate of intraoperative need for vasopressor, 9) incidence of unplanned intensive care unit (ICU) admission, 10) rate of need for rapid response team call, 11) length of stay in ICU, 12) hospital length of stay and 13) incidence of in-hospital mortality (see eTable 2 in Online Supplement for all definitions). These secondary outcomes were also derived from our previously reported primary trial [1].

### Statistical analysis

The original statistical analysis plan for the primary trial is reported elsewhere; while this analysis was not pre-planned, it was conducted in accordance with the original analysis plan. Categorical variables are reported as counts and percentages and compared with Fisher exact tests; continuous variables are reported as median (interquartile range [IQR]) and compared with Wilcoxon rank-sum tests. Patients were analysed according to the group in which they were randomised in the original trial, and the analysis dataset included all patients who were randomised and received general anaesthesia for eligible surgery. The quantity of missing data for the primary outcome was small; therefore, only a complete case analysis was conducted, and no assumption for missing data was made.

The effect of the intervention on the primary outcome is reported as numbers and percentages with risk differences and 95% confidence intervals (CIs), calculated using a generalised linear model with binomial distribution and an identity link function. All other binary outcomes were analysed identically. The effect of the intervention on the length of ICU stay and hospital stay was estimated with generalised linear models considering a Gaussian distribution.

For all outcomes, multivariable analyses were performed using the models described above and incorporating adjustments for independent covariates of age, sex, baseline oxygen saturation (measured by pulse oximetry), body mass index and the Assess Respiratory Risk in Surgical Patients in Catalonia (ARISCAT) score [26]. All covariates were pre-specified based on their known associations with the outcome and according to the original plan. This approach is consistent with previous studies [26, 27]. For the final models, missing data in covariates were imputed by the median. As a sensitivity analysis, a multiple imputation was used to impute missing data in outcomes and covariates (described in the Additional Methods in Online Supplement).

All continuous variables were standardised to improve convergence—the results represent the change in the outcome according to the increase in one standard deviation of the continuous predictor. All analyses were not adjusted for multiplicity, and the confidence intervals (CIs) should not be used to infer definite differences between the groups. The rate of missing data is shown in eTable 3 in the Online Supplement. A two-sided *p* value < 0.05 was considered evidence of statistical significance. All analyses were performed using R software, version 4.0.2 (R Core Team).

## Results

### Patients

From February 2015 to February 2019, 1236 patients were randomised. Of these, 627 were assigned randomly to receive LTVV and 609 patients to receive CTVV. In 30 patients, either the surgery did not proceed, or the anaesthetist declined to participate in the trial (i.e., declined trial protocol ventilation or no arterial line was planned to be placed). These 30 patients were excluded, leaving data from 1206 patients in the primary analysis. A total of 328 patients (27.2%) underwent laparoscopic surgeries, with 158 (48.2%) randomised to LTVV. Data related to the primary outcome were missing for two patients (see eFigure 1 in Online Supplement).

Baseline characteristics and clinical outcomes of the patients are shown in Table 1. The median (IQR) age was 63 (55–71) years, 60.4% were male, and the median (IQR) ARISCAT was 32 (25–31). Within the cohort, 65.8% were classified as at moderate risk for PPCs, and 44.3% were classified as American Society of Anesthesiologists (ASA) 3. The most common comorbidities were hypertension and obesity, respectively. All characteristics were well balanced between the randomisation groups (Table 1).

**Table 1:** Baseline patient characteristics

	Overall (***n*** = 328)	Low Tidal Volume (***n*** = 158)	Conventional Tidal Volume (***n*** = 170)	***p*** value
Age (years)	63 (55–71)	63 (56–71)	62 (55–71)	0.667
Male gender (no. [%])	198 (60.4)	99 (62.7)	99 (58.2)	0.431
Weight (kg)	81.0 (69.8–96.0)	81.0 (68.0–97.0)	80.5 (71.8–95.8)	0.959
BMI (kg/m^2^)^a^	28.1 (24.5–32.6)	28.1 (24.2–32.6)	28.1 (24.8–32.3)	0.739
ARISCAT score (no. / N [%])^b^	32.5 (25.5–41.0)	31.0 (26.0–41.0)	34.0 (23.0–41.0)	0.712
Low risk	60 / 240 (25.0)	25 / 119 (21.0)	35 / 121 (28.9)	0.288
Moderate risk	158 / 240 (65.8)	84 / 119 (70.6)	74 / 121 (61.2)	
High risk	22 / 240 (9.2)	10 / 119 (8.4)	12 / 121 (9.9)	
ASA physical status (no. / N [%])^c^				0.563
1: healthy	26 / 323 (8.0)	10 / 156 (6.4)	16 / 167 (9.6)	
2: mild systemic disease	142 / 323 (44.0)	66 / 156 (42.3)	76 / 167 (45.5)	
3: severe systemic disease	143 / 323 (44.3)	73 / 156 (46.8)	70 / 167 (41.9)	
4: constant threat to life	12 / 323 (3.7)	7 / 156 (4.5)	5 / 167 (3.0)	
Baseline SpO_2_ (%)	97 (96–98)	97 (96–98)	97 (96–98)	0.619
Baseline HCO_3_ (mmol/L)	26 (24.5–28)	26 (24–28)	26 (25–28)	0.291
Baseline haemoglobin (g/dL)	139 (123–150)	141 (127–152)	138 (122–149)	0.083
Baseline creatinine (mg/dL)	0.88 (0.74 − 1.07)	0.89 (0.74 − 1.06)	0.87 (0.72–1.08)	0.615
Comorbidities (no. / N [%])				
Diabetes	63 / 328 (19.2)	28 / 158 (17.7)	35 / 170 (20.6)	0.575
Hypertension	170 / 328 (51.8)	81 / 158 (51.3)	89 / 170 (52.4)	0.912
Obesity^d^	123 / 320 (38.4)	55 / 153 (35.9)	68 / 167 (40.7)	0.421
Coronary artery disease	54 / 328 (16.5)	24 / 158 (15.2)	30 / 170 (17.6)	0.556
Chronic kidney disease	28 / 328 (8.5)	12 / 158 (7.6)	16 / 170 (9.4)	0.693
Chronic liver disease	28 / 328 (8.5)	9 / 158 (5.7)	19 / 170 (11.2)	0.112
Smoking	58 / 328 (17.7)	33 / 158 (20.9)	25 / 170 (14.7)	0.150
Chronic obstructive pulmonary disease	39 / 328 (11.9)	20 / 158 (12.7)	19 / 170 (11.2)	0.734
Asthma	28 / 328 (8.5)	10 / 158 (6.3)	18 / 170 (10.6)	0.235
Obstructive sleep apnoea	38 / 328 (11.6)	16 / 158 (10.1)	22 / 170 (12.9)	0.491
Recent respiratory infection	6 / 328 (1.8)	5 / 158 (3.2)	1 / 170 (0.6)	0.110
Emergency surgery (no. [%])	9 (2.7)	7 / 158 (4.4)	2 (1.2)	0.094

Data are median (25th–75th quartile) or N (%)

*Abbreviations*: *BMI* Body Mass Index, *ARISCAT* Assess Respiratory Risk in Surgical Patients in Catalonia, *ASA* American Society of Anesthesiologists, *HCO*_*3*_ bicarbonate, *SpO*_*2*_ pulse oximetry

^a^Calculated as weight in kilograms divided by height in metres squared

^b^Score range is from 0 to 123; higher scores indicate a higher risk of postoperative pulmonary complications. Patients with scores of 26 or greater are considered at intermediate risk; those with a score greater than 44 are considered at high risk

^c^Scores on the ASA (American Society of Anesthesiologists) physical status classification system ranges from 1 to 6, with higher scores indicating more severe condition

^d^Defined as BMI > 30 kg/m^2^

### Intraoperative procedures

Ventilatory and surgical variables are shown in Table 2.

**Table 2:** Ventilatory and surgical variables in the included patients

	Overall (***n*** = 328)	Low Tidal Volume (***n*** = 158)	Conventional Tidal Volume (***n*** = 170)	***p*** value
Tidal volume				
Absolute (mL)	480 (400–630)	400 (350–450)	610 (520–700)	< 0.001
Adjusted (mL/kg PBW)^a^	8.8 (6.0–10.0)	6.0 (6.0–6.2)	10.0 (9.9–10.1)	< 0.001
PEEP (cmH_2_O)	5 (5–5)	5 (5–5)	5 (5–5)	–
Peak pressure (cmH_2_O)	27 (23–32)	26 (22–30)	29 (24–32)	< 0.001
Respiratory rate (breaths/min)	14 (12–17)	16 (14–18)	12 (10–14)	< 0.001
SpO_2_ (%)	97 (96–98)	97 (95–98)	97 (96–98)	0.144
FiO_2_ (%)	70 (50–96)	70 (50–95)	66.5 (50–99.5)	0.580
etCO_2_ (%)	40 (37–44)	42 (40–47)	39 (36–42)	< 0.001
Arterial blood gas after induction				
pH	7.40 (7.37–7.43)	7.39 (7.35–7.41)	7.42 (7.39–7.45)	< 0.001
PaO_2_ (mmHg)	211 (158–274)	203 (157–272)	219 (164–275)	0.256
PaCO_2_ (mmHg)	41 (38–45)	43 (40–47)	39 (36–42)	< 0.001
HCO_3_ (mmol/L)	25 (24–26)	25 (24–27)	25 (24–26)	0.007
PaO_2_ / FiO_2_ (mmHg)	412 (313–492)	405 (300–481)	417 (339–497)	0.190
Haemoglobin (g/dL)	127 (112–138)	128 (114–139)	125 (110–138)	0.312
Base excess (mEq/L)	1.0 (−0.3–2.0)	0.8 (−0.4–2.0)	1.0 (−0.1–2.0)	0.896
Lactate (mmol/L)	1.1 (0.9–1.4)	1.1 (0.8–1.4)	1.1 (0.9–1.5)	0.197
Arterial blood gas prior to closure				
pH	7.34 (7.30–7.39)	7.32 (7.27–7.36)	7.37 (7.33–7.41)	< 0.001
PaO_2_ (mmHg)	172 (132–219)	168 (132–213)	178 (133–221.8)	0.316
PaCO_2_ (mmHg)	45 (41–52)	50 (43–55)	42 (38–46)	< 0.001
HCO_3_ (mmol/L)	24 (23–25.5)	24 (23–26)	24 (22–25)	0.010
PaO_2_ / FiO_2_ (mmHg)	367 (279–445)	357 (264–438)	371 (290–450)	0.282
Haemoglobin (g/dL)	124 (111–137)	126 (113–140)	122 (110–134)	0.143
Base excess (mEq/L)	–1.0 (−2.4–0.1)	–1.0 (−2.6–0.0)	−0.9 (−2.2–0.5)	0.205
Lactate (mmol/L)	1.1 (0.8–1.5)	1.0 (0.8–1.4)	1.2 (0.9–1.6)	0.039
Duration of surgery (minutes)	185 (140–240)	190 (145–240)	184 (135–238)	0.598
Use of regional anaesthesia (no. / N [%])^b^	59 / 322 (18.3)	29 / 156 (18.6)	30 / 166 (18.1)	0.999
Epidural	0 / 322 (0.0)	0 / 156 (0.0)	0 / 166 (0.0)	–
Spinal opioid	26 / 322 (8.1)	13 / 156 (8.3)	13 / 166 (7.8)	0.999
TAP/abdominal block^c^	22 / 322 (6.8)	12 / 156 (7.7)	10 / 166 (6.0)	0.660
Other^d^	25 / 322 (7.8)	11 / 156 (7.1)	14 / 166 (8.4)	0.682

Data are median (25th–75th quartile) or N (%)

*Abbreviations*: *ABG* arterial blood gas, *etCO*_*2*_ end-tidal carbon dioxide, *FiO*_*2*_ inspired fraction of oxygen, *HCO*_*3*_ bicarbonate, *PaO*_*2*_ partial pressure of oxygen, PaCO_2_ partial pressure of carbon dioxide, *PBW* predicted body weight, *SpO*_*2*_ pulse oximetry, *PEEP* positive end-expiratory pressure

^a^ PBW was calculated as 50 + 0.91 x (height [cm] – 152.4) for men and 45.5 + 0.91 x (height [cm] – 152.4) for women

^b^ In addition to general anaesthesia

^c^ Transversus abdominis plane block (TAP) is defined as block of a peripheral nerve designed to anaesthetise the nerves supplying the anterior abdominal wall (T6 to L1)

^d^ Other: brachial plexus block, femoral nerve block, fascia iliaca block, sciatic nerve block, intercostal nerve block, interpleural catheter, wound catheter

Patients allocated to the LTVV group had lower absolute (400 [350–450] vs. 610 [510–700] mL; absolute difference, − 202.0 [95% CI, − 225.3 to − 178.8]; *p* <  0.001) and adjusted tidal volume (6.0 [6.0–6.2] vs. 10.0 [9.9–10.1] mL/kg PBW; absolute difference, − 3.4 [95% CI, − 3.6 to − 3.1]; *p* <  0.001).

PEEP was similar between the groups In the LTVV group, peak inspiratory pressure was lower (absolute difference, − 2.5 cmH2O [95% CI, − 3.9 to − 1.2]; *p* <  0.001) and respiratory rate was higher (absolute difference, 4.6 breaths/minute [95% CI, 3.9 to 5.3]; *p* <  0.001). SpO_2_ and FiO_2_ were similar between the groups, and etCO_2_ was higher in the LTVV group (absolute difference, 4.2 mmHg [95% CI, 2.9 to 5.5]; *p* <  0.001).

After induction, pH was lower and PaCO2 was higher in the LTVV group. Except for bicarbonate (where the difference was not clinically significant), all other laboratory tests were similar between the groups, including the PaO_2_ (see Table 2). The differences in the arterial blood gas prior to closure were similar to those found after induction. The duration of surgery was similar between the groups, as was the use of regional anaesthesia.

### Primary outcome

A total of 52 of 157 patients (33.1%) assigned to LTVV and 72 of 169 (42.6%) assigned to conventional tidal volume developed PPCs within 7 days (unadjusted absolute difference, − 9.48 [95% CI, − 19.86 to 1.05]; *p* = 0.076) (see Table 3). After adjusting for pre-specified confounders, patients in the LTVV group had a lower incidence of the primary outcome than patients in the conventional group (adjusted absolute difference, − 10.36 [95% CI, − 20.52 to − 0.20]; *p* = 0.046).

**Table 3:** Primary and secondary outcomes in the included patients

	Overall (***n*** = 328)	Low Tidal Volume (***n*** = 158)	Conventional Tidal Volume (***n*** = 170)	Unadjusted Analysis		Adjusted Analysis*	
				Absolute Difference (95% CI)	***p*** value	Absolute Difference (95% CI)	***p*** value
PPC within 7 days	124 / 326 (38.0)	52 / 157 (33.1)	72 / 169 (42.6)	−9.48 (− 19.86 to 1.05)^a^	0.076	−10.36 (− 20.52 to − 0.20)^a^	0.046
Pneumonia	12 / 327 (3.7)	6 / 158 (3.8)	6 / 169 (3.6)	0.25 (− 4.02 to 4.64)^a^	0.906	0.12 (−3.98 to 4.22)^a^	0.954
Respiratory failure	54 / 327 (16.5)	21 / 158 (13.3)	33 / 169 (19.5)	−6.24 (−14.26 to 1.81)^a^	0.126	−6.71 (− 14.66 to 1.25)^a^	0.098
Pleural effusion	31 / 326 (9.5)	15 / 158 (9.5)	16 / 168 (9.5)	− 0.03 (− 6.48 to 6.49)^a^	0.993	−0.31 (−6.61 to 5.99)^a^	0.924
Atelectasis	84 / 327 (25.7)	36 / 157 (22.9)	48 / 170 (28.2)	−5.31 (−14.70 to 4.18)^a^	0.270	−6.09 (−15.40 to 3.23)^a^	0.199
Pneumothorax	0 / 327 (0.0)	0 / 158 (0.0)	0 / 169 (0.0)	–	–	–	–
Bronchospasm	5 / 327 (1.5)	2 / 158 (1.3)	3 / 169 (1.8)	−0.51 (−3.57 to 2.48)^a^	0.706	− 0.51 (− 3.20 to 2.19)^a^	0.711
Pulmonary congestion	6 / 327 (1.8)	2 / 158 (1.3)	4 / 169 (2.4)	−1.10 (−4.43 to 2.02)^a^	0.454	−1.20 (− 4.14 to 1.75)^a^	0.424
Unplanned NIV or IMV	7 / 327 (2.1)	3 / 158 (1.9)	4 / 169 (2.4)	−0.47 (−3.93 to 2.98)^a^	0.769	−0.43 (−3.55 to 2.69)^a^	0.786
PPC during hospital stay	128 / 324 (39.5)	53 / 157 (33.8)	75 / 167 (44.9)	−11.15 (−21.60 to −0.52)^a^	0.039	− 12.13 (− 22.35 to − 1.91)^a^	0.020
Pulmonary embolism	2 / 324 (0.6)	1 / 158 (0.6)	1 / 166 (0.6)	0.03 (−2.16 to 2.31)^a^	0.972	0.07 (−1.63 to 1.77)^a^	0.933
Acute respiratory distress syndrome	0 / 324 (0.0)	0 / 158 (0.0)	0 / 166 (0.0)	–	–	–	–
SIRS	1 / 326 (0.3)	0 / 157 (0.0)	1 / 169 (0.6)	−0.60 (−1.80 to 0.62)^a^	0.336	−0.58 (−1.78 to 0.63)^a^	0.347
Sepsis	11 / 327 (3.4)	7 / 158 (4.4)	4 / 169 (2.4)	2.06 (−1.92 to 6.45)^a^	0.305	2.22 (−1.72 to 6.16)^a^	0.269
Acute kidney injury	24 / 228 (10.5)	11 / 115 (9.6)	13 / 113 (11.5)	−1.94 (− 10.14 to 6.15)^a^	0.633	− 1.60 (−9.61 to 6.42)^a^	0.695
Risk	18 / 228 (7.9)	8 / 115 (7.0)	10 / 113 (8.8)				
Injury	1 / 228 (0.4)	1 / 115 (0.9)	0 / 113 (0.0)				
Failure	5 / 228 (2.2)	2 / 115 (1.7)	3 / 113 (2.7)				
Wound infection	6 / 325 (1.8)	3 / 158 (1.9)	3 / 167 (1.8)	0.10 (−3.13 to 3.44)^a^	0.945	−0.09 (−3.00 to 2.83)^a^	0.952
Intraoperative need of vasopressor	269 / 322 (83.5)	130 / 156 (83.3)	139 / 166 (83.7)	−0.40 (−8.59 to 7.72)^a^	0.923	−0.80 (−8.85 to 7.25)^a^	0.845
Unplanned ICU admission	15 / 320 (4.7)	8 / 155 (5.2)	7 / 165 (4.2)	0.92 (−3.85 to 5.87)^a^	0.698	1.03 (−3.62 to 5.69)^a^	0.663
Need for MET call	26 / 327 (8.0)	12 / 158 (7.6)	14 / 169 (8.3)	−0.69 (−6.66 to 5.33)^a^	0.818	−0.78 (−6.62 to 5.06)^a^	0.793
Length of stay							
In ICU (hours)	27.3 ± 33.6	29.4 ± 31.8	26.0 ± 35.1	3.36 (−15.09 to 21.82)^b^	0.716	6.51 (−12.04 to 25.06)^b^	0.484
Median (IQR)	15 (13 to 27)	17 (12 to 28)	15 (13 to 20)				
In hospital (days)	6.7 ± 6.0	7.0 ± 6.4	6.4 ± 5.7	0.64 (−0.66 to 1.96)^b^	0.333	0.54 (−0.72 to 1.81)^b^	0.399
Median (IQR)	5 (3 to 8)	5 (3 to 8)	4 (3 to 7)				
In-hospital mortality	5 / 328 (1.5)	3 / 158 (1.9)	2 / 170 (1.2)	0.72 (−1.95 to 3.39)^a^	0.595	0.78 (− 1.86 to 3.42)^a^	0.562

Data are median (25th–75th quartile) or no. / N (%)

*Abbreviations*: *CI* confidence interval, *ICU* intensive care unit, *MET* medical emergency team, *PPCs* postoperative pulmonary complications, *NIV* non-invasive ventilation, *IMV* invasive mechanical ventilation, *SIRS* Systemic Inflammatory Response Syndrome

*All models adjusted by age, sex, baseline SpO_2_, body mass index

^a^Effect estimate is risk difference from a generalised linear model considering a binomial distribution

^b^Effect estimate is mean difference from a generalised linear model considering a Gaussian distribution

A total of 53 of 157 patients (33.8%) assigned to LTVV and 75 of 167 (44.9%) assigned to conventional tidal volume developed PPCs during their hospital stay (unadjusted absolute difference, − 11.15 [95% CI, − 21.60 to − 0.52]; *p* = 0.039; adjusted absolute difference, − 12.13 [95% CI, − 22.35 to − 1.91]; *p* = 0.020) (see Table 3). All other secondary outcomes were similar for both groups before and after adjustment for confounders.

### Sensitivity analysis

The results obtained after multiple imputations for missing data in outcomes and covariates are shown in eTable 4 in v Online Supplement. After multiple imputations and adjustment for confounders, patients in the LTVV group had a lower incidence of the primary outcome (adjusted absolute difference, − 10.58 [95% CI, − 20.83 to − 0.32]; *p* = 0.043) and PPCs during hospital stay (adjusted absolute difference, − 11.70 [95% CI, − 21.87 to − 1.53]; *p* = 0.024).

## Discussion

### Key findings

In this post-hoc analysis of a randomised controlled trial of adult patients undergoing major laparoscopic surgery, intraoperative mechanical ventilation with low tidal volumes was associated with a significant reduction in post-operative pulmonary complications compared with conventional tidal volume ventilation.

### Relationship to previous studies

In our recent randomised trial of patients undergoing major surgery, we found no overall benefit of LTVV relative to conventional tidal volume ventilation when PEEP was equalised between groups [1]. The results of this post-hoc analysis of patients undergoing laparoscopic surgery suggest that this subgroup may represent a unique subset of patients that might benefits specifically from a low tidal volume strategy. Such differences may reflect the physiological effects of different operative interventions on respiratory function, including the use of pneumoperitoneum and its associated raised intraabdominal pressure, duration of surgery, variations in patient position during surgery and the magnitude of postoperative pain [15–17]. Further suggestion of a difference in patient–ventilator interaction during laparoscopic surgery was apparent in another large randomised controlled trial that focused on the effect of a high PEEP versus a low PEEP strategy when combined with LTVV in obese patients during surgery [28]. This study did not demonstrate a difference in PPCs in a combined population of open and laparoscopic abdominal surgery patients. However, in the subgroup of patients undergoing laparoscopic surgery, a high PEEP strategy was associated with a numerically lower rate of PPCs within 7 days [28]. A subsequent meta-analysis by Campos also suggested laparoscopic patients represented a unique group which may benefit from a high PEEP strategy (Ref Campos et al).

Previous studies of the effect of LTVV during laparoscopic surgery have been small, underpowered, and focused on physiological rather than clinical outcomes; further, they have been often confined to highly specific operative procedures [21–24]. Despite these limitations, such studies supported the biological plausibility of the potential benefits of a ventilation strategy that incorporates a low tidal volume in patients receiving laparoscopic abdominal surgery. These benefits included improved gas exchange and respiratory mechanics and a reduction in atelectasis [21–24].

Our results could be explained by the physiological effects of the pneumoperitoneum on transpulmonary pressure (particularly in the context of moderate PEEP as in our trial). When only moderate PEEP is applied to patients with raised intrabdominal pressure, prevailing conditions are established which may promote suboptimal recruitment and reduced functional residual capacity. When a higher tidal volume is then applied, it is plausible that patients would be subjected to higher lung strain (defined as a ratio of tidal volume to end expiratory lung volume). Increased lung strain (and its surrogate driving pressure) has been identified as an important factor in the pathogenesis of ventilator lung injury [29–32].

### Strengths and limitations

Our study is the largest investigation of LTVV during laparoscopic surgery. It analysed a pre-defined cohort of significance. It used an outcome that as assessed by observers blinded to the intervention. It adjusted findings in the laparoscopic group for any imbalances in relevant confounders. Moreover, it applied imputation analysis to test the robustness of its finding. Finally, it was the first to assess the effect of LTVV in isolation. In this regard, previous studies of patients undergoing laparoscopic surgeries have focused commonly on LTVV combined with differences in PEEP and recruitment manoeuvres, which have been proposed to oppose the physiological effects of the pneumoperitoneum [19, 20, 24]. Our results suggest benefits when LTVV is applied in isolation.

This study has several limitations. Importantly, this was a post-hoc analysis of a single centre study; therefore, it is subject to all the limitations intrinsic to such a study type. However, this study did include a diverse range of patients and laparoscopic procedures. In addition, blinding of clinical staff was not possible primarily due to the nature of the intervention. However, scoring of the clinical outcomes was determined by blinded observers. Unfortunately, due to limitations of the ventilators used in our centre at the time of our primary trial we were unable to measure plateau pressure and estimate driving pressure (as a surrogate of lung strain) during surgery. Notably, the respiratory management after the intraoperative intervention period was not protocolised. However, there are no current consensus guidelines to guide such management. Therefore, we designed this trial with a pragmatic approach. Furthermore, the use of the primary composite outcome implies equivalence of each of its components and the severity of the complication was not qualified in the results. However, this is consistent with the approach used in our overarching trial. This is also congruous with other major trials of intraoperative ventilation and PPCs [1, 2, 28, 33]. Consequently, such complications were included regardless of severity. Nevertheless, we would suggest that even minor PPCs would be considered important. Finally, chest x-rays were performed in this trial only when clinically indicated (as determined by the treating staff) and were not systematically evaluated. However, this limitation would not have been a likely source of bias. Finally, we did not record the time from surgery to the onset of a postoperative pulmonary complication. However, would note this time frame is consistent with previously suggested definitions [34].

In conclusion, this post-hoc analysis of a large, randomised trial of LTVV during major laparoscopic found that LTVV was associated with a significantly reduced risk of PPCs compared to conventional tidal volume ventilation, when PEEP was applied equally between both groups. Given these results, further evaluation of the ideal ventilation strategy during laparoscopic surgery, is warranted.

## Supplementary Information

**Additional file 1: **Additional methods. **eTable 1.** Definition of the primary outcome. **eTable 2.** Definitions of the secondary outcomes. **eTable 3.** Rate of Missing Data. **eTable 4.** Primary and Secondary Outcomes in the Included Patients After Multiple Imputation. **eFigure 1.** Study Flowchart.

## Data Availability

We accept participation in a data sharing arrangement on reasonable request where specifically relevant to the results of this study. These can be available from the corresponding author on reasonable request.

## Abbreviations

ASA: American Society of Anesthesiologists
ABG: Arterial blood gas
ARISCAT: Assess Respiratory Risk in Surgical Patients in Catalonia
HCO_3_: Bicarbonate
BMI: Body mass index
COPD: Chronic obstructive pulmonary disease
CI: Confidence interval
etCO_2_: End-tidal carbon dioxide
FiO_2_: Inspired fraction of oxygen
ICU: Intensive care unit
IQR: Interquartile range
IMV: Invasive mechanical ventilation
LTVV: Low tidal volume ventilation
MET: Medical emergency team
NIV: Non-invasive ventilation
PaCO_2_: Partial pressure of carbon dioxide
PaO_2_: Partial pressure of oxygen
PEEP: Positive end-expiratory pressure
PPCs: Postoperative pulmonary complications
PBW: Predicted body weight
SpO_2_: Pulse oximetry
TAP: Transversus abdominis plane block
