# Supplementary material for: Low tidal volume ventilation for patients undergoing laparoscopic surgery: a secondary analysis of a randomised clinical trial
Source: BMC Anesthesiol. 2023 Mar 7;23:71. doi: 10.1186/s12871-023-01998-1 (PMC9990198; doi:10.1186/s12871-023-01998-1)
Supplement: Supplementary file 1 — Additional file 1: Additional methods. eTable 1. Definition of the primary outcome. eTable 2. Definitions of the secondary outcomes. eTable 3. Rate of Missing Data. eTable 4. Primary and Secondary Outcomes in the Included Patients After Multiple Imputation. eFigure 1. Study Flowchart. [file 12871_2023_1998_MOESM1_ESM.docx]

**Low Tidal Volume Ventilation for Patients Undergoing Laparoscopic Surgery - A secondary analysis of a randomized clinical trial**

ONLINE SUPPLEMENT

**ADDITIONAL METHODS**

*Multiple imputation*

For patients with missing values in outcomes and covariates, multiple imputation by chained equation method (MICE) was used. By leveraging known patient characteristics and accounting for uncertainty in the multiple estimations of missing values, multiple imputation preserves sample size and reduces bias while examining association between variables. Before imputation, the percentage of missingness in the variables was assessed.

The following variables were considered as predictors in the multiple imputation model: age, sex, baseline SpO_2_, body mass index, total ARISCAT, ASA score, presence of hypertension, emergency surgery, duration of surgery, and all primary and secondary outcomes.

The lower and upper cut-offs of imputed values respected the upper and lower limits of the original variables in patients without missing data. Imputation was performed using predictive mean matching for continuous variables, logistic regression for binary variables and polytomous regression for categorical variables with more than two categories. A model in five imputed datasets with 50 iterations was used. After imputation all models were repeated and results combined across imputations using Rubin’s rule.

| **eTable 1 – Definition of the primary outcome** | |
| --- | --- |
|  | **Definition** |
| Pneumonia | Defined as need of antibiotics for a suspected respiratory infection and one or more of the following criteria: new or changed sputum, new or changed lung opacities, fever and/or white blood cell count > 12x10^9^/L |
| Bronchospasm | Defined as newly detected expiratory wheeze treated with bronchodilators |
| Atelectasis | Defined as lung opacification with a shift of the mediastinum, hilum or hemidiaphragm toward the affected area, and compensatory over-inflation in the adjacent non-atelectatic lung |
| Pulmonary congestion | Defined as clinical signs of congestion, including dyspnoea, oedema, rales, and jugular venous distention, with or without chest x–ray demonstrating increase in vascular markings and diffuse alveolar interstitial infiltrates |
| Respiratory failure | Defined as a postoperative PaO_2_ < 60 mmHg on room air, a PaO_2_ / FiO_2_ ratio < 300 mmHg or arterial oxyhaemoglobin saturation measured with pulse oximetry < 90% and requiring oxygen therapy |
| Pleural effusion | Defined as chest radiograph demonstrating blunting of the costophrenic angle, loss of sharp silhouette of the ipsilateral hemidiaphragm in upright position, evidence of displacement of adjacent anatomical structures or (in supine position) a hazy opacity in one hemithorax with preserved vascular shadows |
| Pneumothorax | Defined as air in the pleural space with no vascular bed surrounding the visceral pleura |
| Requirement for mechanical ventilation | Defined as unplanned need of non–invasive or invasive ventilation |

| **eTable 2 – Definitions of the secondary outcomes** | |
| --- | --- |
| **Outcome** | **Definition** |
| Postoperative pulmonary complications during hospital stay | As defined in eTable 1 but considering the whole hospital stay |
| Pulmonary embolism | Presence of segmental or subsegmental pulmonary embolus diagnosed on computerized tomography or high probability pulmonary embolus on ventilator-perfusion scan |
| Acute respiratory distress syndrome | According to Berlin definition |
| SIRS | Two or more of the following:   - Temperature < 36 °C or > 38 °C; or - Heart rate > 90 beats per minute; or - Respiratory rate) > 20 breaths per minute; or, PaCO_2_ < 32 mmHg; or - White blood cell count < 4000 cells/mm³ or > 12000 cells/mm³; or more than 10% immature neutrophils (band forms). |
| Sepsis | SIRS criteria plus a definitive or presumed infectious foci |
| Acute kidney injury | According to RIFLE criteria:   - Risk: 1.5-fold increase in the serum creatinine, GFR decrease by 25%, or urine output < 0.5 mL/kg per hour for 6 hours; or - Injury: Two-fold increase in the serum creatinine, or GFR decrease by 50%, or urine output < 0.5 mL/kg per hour for 12 hours; or - Failure: Three-fold increase in the serum creatinine, or GFR decrease by 75%, or urine output of < 0.3 mL/kg per hour for 24 hours, or no urine output (anuria) for 12 hours; or - Loss: Complete loss of kidney function (e.g., need for renal replacement therapy) for more than four weeks; or - End-stage kidney disease: Complete loss of kidney function (e.g., need for renal replacement therapy) for more than three months. |
| Wound infection | Superficial or deep surgical site infection according to CDC definition in the first seven days |
| Intraoperative need of vasopressor | Defined as the need of aramine, norepinephrine, ephedrine, phenylephrine, epinephrine and/or vasopressin in the intraoperative period |
| Unplanned ICU admission | Any admission that was not previously planned in the plan of care |
| Need for rapid response team call | Any call for the rapid response team while in the ward |
| ICU length of stay | Days from ICU admission to ICU discharge |
| Hospital length of stay | Days from hospital admission to hospital discharge |
| Hospital mortality | Death from any cause during hospital stay |
| *ICU: intensive care unit; SIRS: systemic inflammatory response syndrome; CDC: Centers for Disease Control; GFR: glomerular filtration rate* | |

| **eTable 3 - Rate of Missing Data** | | | |
| --- | --- | --- | --- |
|  | **Overall**  **(*n* = 328)** | **Low Tidal Volume**  **(*n* = 158)** | **Conventional Tidal Volume**  **(*n* = 170)** |
| Age | 0 (0.0) | 0 (0.0) | 0 (0.0) |
| Gender | 0 (0.0) | 0 (0.0) | 0 (0.0) |
| Weight | 1 (0.3) | 1 (0.6) | 0 (0.0) |
| Body mass index | 8 (2.4) | 5 (3.2) | 3 (1.8) |
| ARISCAT score | 88 (26.8) | 39 (24.7) | 49 (28.8) |
| ASA score | 5 (1.5) | 2 (1.3) | 3 (1.8) |
| Baseline SpO_2_ | 4 (1.2) | 2 (1.3) | 2 (1.2) |
| Baseline HCO_3_ | 9 (2.7) | 4 (2.5) | 5 (2.9) |
| Baseline hemoglobin | 4 (1.2) | 1 (0.6) | 3 (1.8) |
| Baseline creatinine | 95 (29.0) | 40 (25.3) | 55 (32.4) |
| Diabetes | 0 (0.0) | 0 (0.0) | 0 (0.0) |
| Hypertension | 0 (0.0) | 0 (0.0) | 0 (0.0) |
| Coronary artery disease | 0 (0.0) | 0 (0.0) | 0 (0.0) |
| Chronic kidney disease | 0 (0.0) | 0 (0.0) | 0 (0.0) |
| Chronic liver disease | 0 (0.0) | 0 (0.0) | 0 (0.0) |
| Smoking | 0 (0.0) | 0 (0.0) | 0 (0.0) |
| Chronic obstructive pulmonary disease | 0 (0.0) | 0 (0.0) | 0 (0.0) |
| Asthma | 0 (0.0) | 0 (0.0) | 0 (0.0) |
| Obstructive sleep apnea | 0 (0.0) | 0 (0.0) | 0 (0.0) |
| Obesity | 8 (2.4) | 5 (3.2) | 3 (1.8) |
| Recent respiratory infection | 0 (0.0) | 0 (0.0) | 0 (0.0) |
| Emergency surgery | 0 (0.0) | 0 (0.0) | 0 (0.0) |
| Absolute tidal volume | 6 (1.8) | 2 (1.3) | 4 (2.4) |
| Adjusted tidal volume | 13 (4.0) | 6 (3.8) | 7 (4.1) |
| PEEP | 0 (0.0) | 0 (0.0) | 0 (0.0) |
| Peak pressure | 7 (2.1) | 3 (1.9) | 4 (2.4) |
| Respiratory rate | 7 (2.1) | 2 (1.3) | 5 (2.9) |
| SpO_2_ | 6 (1.8) | 2 (1.3) | 4 (2.4) |
| FiO_2_ | 7 (2.1) | 3 (1.9) | 4 (2.4) |
| etCO_2_ | 7 (2.1) | 2 (1.3) | 5 (2.9) |
| Arterial blood gas after induction |  |  |  |
| pH | 20 (6.1) | 9 (5.7) | 11 (6.5) |
| PaO_2_ | 19 (5.8) | 8 (5.1) | 11 (6.5) |
| PaCO_2_ | 20 (6.1) | 8 (5.1) | 12 (7.1) |
| HCO_3_ | 24 (7.3) | 10 (6.3) | 14 (8.2) |
| PaO_2_ / FiO_2_ | 28 (8.5) | 11 (7.0) | 17 (10.0) |
| Hemoglobin | 19 (5.8) | 8 (5.1) | 11 (6.5) |
| Base excess | 23 (7.0) | 10 (6.3) | 13 (7.6) |
| Lactate | 34 (10.4) | 14 (8.9) | 20 (11.8) |
| Arterial blood gas prior to closure |  |  |  |
| pH | 33 (10.1) | 18 (11.4) | 15 (8.8) |
| PaO_2_ | 33 (10.1) | 17 (10.8) | 16 (9.4) |
| PaCO_2_ | 31 (9.5) | 16 (10.1) | 15 (8.8) |
| HCO_3_ | 33 (10.1) | 18 (11.4) | 15 (8.8) |
| PaO_2_ / FiO_2_ | 40 (12.2) | 20 (12.7) | 20 (11.8) |
| Hemoglobin | 30 (9.1) | 16 (10.1) | 14 (8.2) |
| Base excess | 35 (10.7) | 19 (12.0) | 16 (9.4) |
| Lactate | 44 (13.4) | 22 (13.9) | 22 (12.9) |
| Duration of surgery | 7 (2.1) | 1 (0.6) | 6 (3.5) |
| Use of regional anesthesia | 6 (1.8) | 2 (1.3) | 4 (2.4) |
| Epidural | 6 (1.8) | 2 (1.3) | 4 (2.4) |
| Spinal opioid | 6 (1.8) | 2 (1.3) | 4 (2.4) |
| TAP/abdominal block | 6 (1.8) | 2 (1.3) | 4 (2.4) |
| Other | 6 (1.8) | 2 (1.3) | 4 (2.4) |
| PPC within 7 days | 2 (0.6) | 1 (0.6) | 1 (0.6) |
| Pneumonia | 1 (0.3) | 0 (0.0) | 1 (0.6) |
| Respiratory failure | 1 (0.3) | 0 (0.0) | 1 (0.6) |
| Pleural effusion | 2 (0.6) | 0 (0.0) | 2 (1.2) |
| Atelectasis | 1 (0.3) | 1 (0.6) | 0 (0.0) |
| Pneumothorax | 1 (0.3) | 0 (0.0) | 1 (0.6) |
| Bronchospasm | 1 (0.3) | 0 (0.0) | 1 (0.6) |
| Pulmonary congestion | 1 (0.3) | 0 (0.0) | 1 (0.6) |
| Unplanned NIV or IMV | 1 (0.3) | 0 (0.0) | 1 (0.6) |
| PPC during hospital stay | 4 (1.2) | 1 (0.6) | 3 (1.8) |
| Pulmonary embolism | 4 (1.2) | 0 (0.0) | 4 (2.4) |
| Acute respiratory distress syndrome | 4 (1.2) | 0 (0.0) | 4 (2.4) |
| SIRS | 2 (0.6) | 1 (0.6) | 1 (0.6) |
| Sepsis | 1 (0.3) | 0 (0.0) | 1 (0.6) |
| Acute kidney injury | 100 (30.5) | 43 (27.2) | 57 (33.5) |
| Wound infection | 3 (0.9) | 0 (0.0) | 3 (1.8) |
| Intraoperative need of vasopressor | 6 (1.8) | 2 (1.3) | 4 (2.4) |
| Unplanned ICU admission | 8 (2.4) | 3 (1.9) | 5 (2.9) |
| Need for MET call | 1 (0.3) | 0 (0.0) | 1 (0.6) |
| ICU length of stay | 271 (82.6) | 136 (86.1) | 135 (79.4) |
| Hospital length of stay | 0 (0.0) | 0 (0.0) | 0 (0.0) |
| Hospital mortality | 0 (0.0) | 0 (0.0) | 0 (0.0) |
| Abbreviations: ARISCAT: Assess Respiratory Risk in Surgical Patients in Catalonia; ASA: American Society of Anesthesiology; COPD: chronic obstructive pulmonary disease; HCO_3_: bicarbonate; ICU: intensive care unit; SpO_2_: pulse oximetry; ABG: arterial blood gas; etCO_2_: end-tidal carbon dioxide; FiO_2_: inspired fraction of oxygen; HCO_3_: bicarbonate; PaO_2_: partial pressure of oxygen. PaCO_2_: partial pressure of carbon dioxide; PBW: predicted body weight; SpO_2_: pulse oximetry; PEEP: positive-end expiratory pressure; MET: medical emergency team; PPC: postoperative pulmonary complications; NIV: non-invasive ventilation; IMV: invasive mechanical ventilation. | | | |

| **eTable 4 - Primary and Secondary Outcomes in the Included Patients After Multiple Imputation** | | |
| --- | --- | --- |
|  | **Adjusted Analysis After Multiple Imputation*** | |
|  | **Absolute Difference**  **(95% CI)** | ***p* value** |
| PPC within 7 days | -10.58 (-20.83 to -0.32)^a^ | 0.043 |
| Pneumonia | -0.12 (-4.33 to 4.08)^a^ | 0.951 |
| Respiratory failure | -7.01 (-15.03 to 1.00)^a^ | 0.086 |
| Pleural effusion | -0.56 (-6.93 to 5.80)^a^ | 0.861 |
| Atelectasis | -5.63 (-15.00 to 3.72)^a^ | 0.237 |
| Bronchospasm | -1.08 (-4.03 to 1.85)^a^ | 0.467 |
| Pulmonary congestion | -1.64 (-4.82 to 1.53) | 0.309 |
| Unplanned NIV or IMV | -0.64 (-3.94 to 2.65)^a^ | 0.700 |
| PPC during hospital stay | -11.70 (-21.87 to -1.53)^a^ | 0.024 |
| Pulmonary embolism | -0.34 (-2.38 to 1.69)^a^ | 0.737 |
| SIRS | -0.70 (-2.14 to 0.74)^a^ | 0.336 |
| Sepsis | 1.77 (-2.31 to 5.87)^a^ | 0.394 |
| Acute kidney injury | -2.42 (-9.95 to 5.10)^a^ | 0.526 |
| Wound infection | -0.31 (-3.41 to 2.79)^a^ | 0.844 |
| Intraoperative need of vasopressor | -0.64 (-8.62 to 7.32)^a^ | 0.873 |
| Unplanned ICU admission | 0.88 (-3.75 to 5.53)^a^ | 0.706 |
| Need for MET call | -0.86 (-6.79 to 5.05)^a^ | 0.773 |
| Length of stay |  |  |
| In ICU, hours | 6.26 (-6.01 to 18.53)^b^ | 0.266 |
| In hospital, days | 0.57 (-0.68 to 1.84)^b^ | 0.370 |
| In–hospital mortality | 0.83 (-1.78 to 3.44)^a^ | 0.533 |
| Data are median (quartile 25^th^ - quartile 75^th^) or no. / N (%).  Abbreviations: CI: confidence interval; ICU: intensive care unit; MET: medical emergency team; PPC: postoperative pulmonary complications; NIV: non-invasive ventilation; IMV: invasive mechanical ventilation.  * All models adjusted by age, sex, baseline SpO_2_, body mass index and total ARISCAT.  ^a^ effect estimate is risk difference from a generalized linear model considering a binomial distribution  ^b^ effect estimate is mean difference from a generalized linear model considering a Gaussian distribution | | |

**eFigure 1 – Study Flowchart**

**
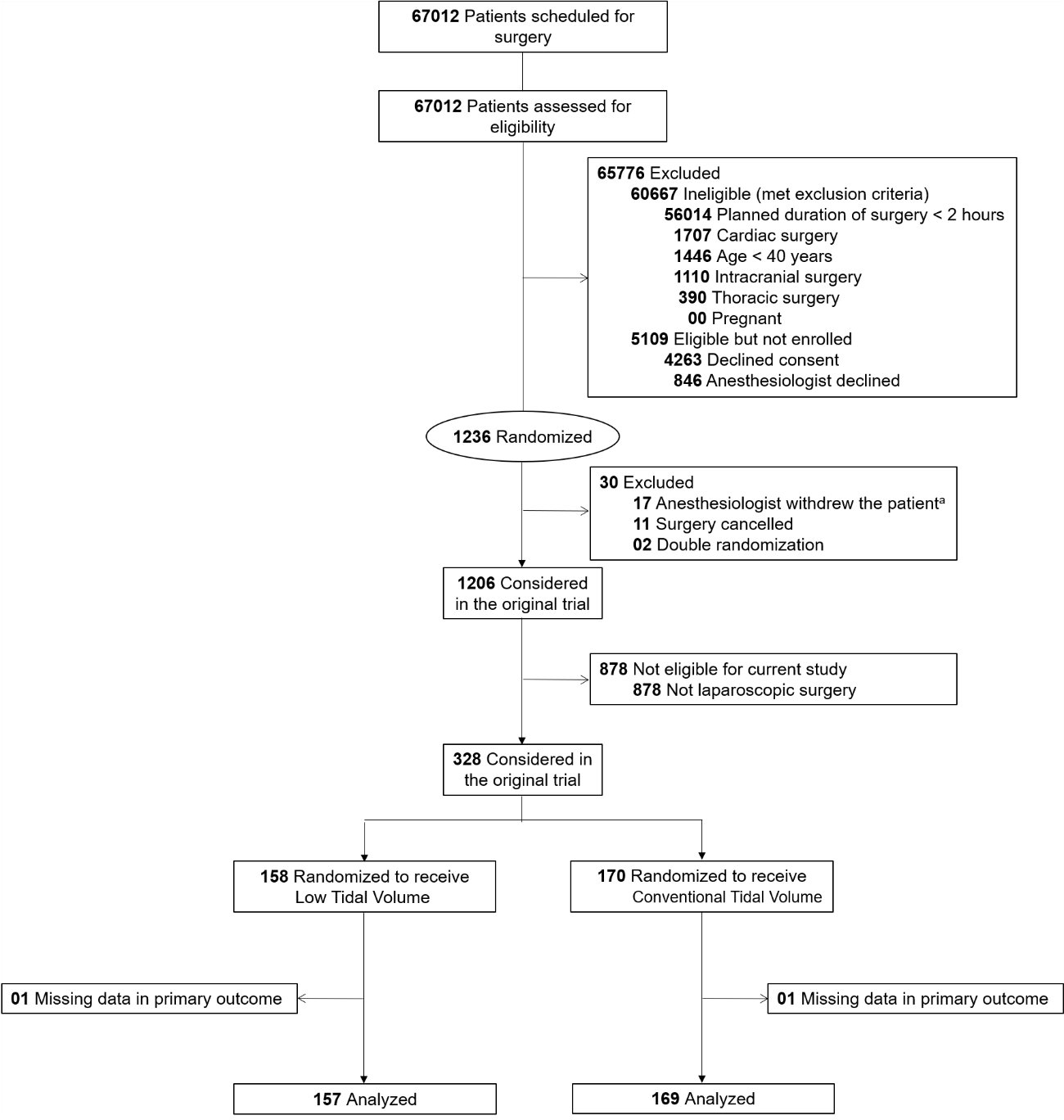
**

^a^ Patients withdrew after randomization by the anesthesiologist (due to lack of clinical equipoise or when an arterial line was not inserted). Reasons for anesthesiologist’s decline were not collected
